# Limonene Modulates Event‐Related Potentials in a 2‐Back Working Memory Task

**DOI:** 10.1002/brb3.71679

**Published:** 2026-08-17

**Authors:** Kaori Tamura, Keitaro Yamashita, Taiki Nishimura, Toko Tatsumoto, Takumi Yano, Yuko Ohno, Shin'ichi Yoshimura, Mitsuo Tonoike

**Affiliations:** ^1^ Department of Basic Sciences, Faculty of Engineering Kyushu Institute of Technology, Kitakyushu, Japan; ^2^ Department of Information and Systems Engineering, Faculty of Information Engineering Fukuoka Institute of Technology, Fukuoka, Japan; ^3^ Department of Information and Systems Engineering, Graduate School of Engineering Fukuoka Institute of Technology, Fukuoka, Japan; ^4^ Department of Mechanical and Control Engineering Kyushu Institute of Technology, Kitakyushu, Japan; ^5^ Division of Health Sciences, Graduate School of Medicine The University of Osaka, Suita, Japan; ^6^ Department of Systems Innovation, Graduate School of Engineering Science The University of Osaka, Suita, Japan

**Keywords:** limonene, multimodal process, P300 amplitudes, working memory

## Abstract

**Introduction:**

Olfactory inputs can influence visual cognition. However, it remains unclear whether such effects are driven by the chemical properties of odorants or by mood‐related associations, especially under conditions of high cognitive demand. This study examined whether limonene, a key citrus odorous compound, modulates cognitive processing during a visual 2‐back task under different levels of perceptual load.

**Methods:**

We developed a 2‐back working memory task using color lightness discrimination. The task included both low‐load (orange) and high‐load (gray) conditions. Participants wore a non‐woven mask impregnated with the odor solution and performed the task under two odor conditions: limonene exposure and no odor. An electroencephalogram was recorded to evaluate P300 amplitude at central (Cz) and parietal (Pz) electrodes.

**Results:**

Behavioral results, analyzed using signal detection theory, showed that the correct response rate and sensitivity index (*d*′) were higher for orange than for gray stimuli, regardless of the odor condition. Event‐related potential (ERP) analysis revealed a significant interaction; under the limonene exposure condition, P300 amplitudes were reduced for gray stimuli, but remained stable for orange stimuli.

**Conclusions:**

Our findings indicate that limonene modulates neural markers associated with attentional and decision‐related processing, particularly under high cognitive demand, without clear effects on behavioral performance. The observed P300 suppression suggests that this component may be sensitive to decreases in attentional allocation that behavioral measures failed to detect. Moreover, these results replicate and extend previous findings of limonene‐induced P300 suppression, supporting the reproducibility of limonene's modulatory effect on neural markers of cross‐modal attentional processing.

## Graphical abstract

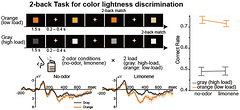

## 1 Introduction

A growing body of evidence suggests that olfactory input can modulate visual processing at both behavioral and neural levels. For example, odor stimuli affect visual attention, perception, and memory even when the odors are not semantically related to visual information. These findings suggest that olfaction and vision are more closely linked than previously thought and that cross‐modal interactions can play an important role in cognitive processing (Gottfried and Dolan 2003; Hörberg et al. 2020; Rekow et al. 2022; Tsushima et al. 2021).

Despite these insights, little is known about how olfactory input affects working memory and attention under a high cognitive load. Our early findings demonstrated that decanal, a major compound in grapefruit peel, modulates visual working memory performance and the P300 amplitude (Tamura et al. 2018). Specifically, decanal reduced performance for orange color stimuli and decreased the P300 amplitudes during the retrieval. However, that study did not assess mood states in detail, leaving it unclear whether the observed modulation arose from the chemical properties of the odorous compound or emotional factors.

Recent behavioral studies have further suggested that olfactory stimulation can influence working memory. For example, Ueda et al. tested three types of essential oil inhalation and reported that lemon essential oil inhalation improved performance in a 2‐back letter memory task after participants had adapted to the odor (Ueda et al. 2023). Notably, the degree and direction of the effect varied by type of essential oil. Additionally, recent evidence on event‐related potentials (ERP) suggests that brief olfactory stimulation can influence memory encoding, as demonstrated by sustained neural activity following odor cues (Tarrida et al. 2025). Another study showed that the effects of aversive olfactory distraction depend on working memory load, and identified neural activity associated with the suppression of distraction under high‐load conditions (Weigard et al. 2021). Although these findings support the idea that odorous compounds can modulate memory‐related cognition, they do not clearly determine whether such effects are primarily driven by mood induction or by the specific chemical properties of the odorous compound itself.

To clarify this distinction, our recent study directly compared the effects of limonene and lemon essential oils in a visual attention task (Tamura et al. 2025). We observed that limonene, a prominent component of citrus peels, reduced the P300 amplitude during a visual oddball task. In contrast, lemon essential oil, which contains limonene, did not significantly suppress the P300 amplitude. The subjective pleasantness ratings were not significantly different between limonene and lemon essential oils. P300 is an ERP component that has been linked to attention, stimulus evaluation, and memory‐related operations (Polich 2007). Therefore, these results suggest that limonene may suppress attentional processes through a compound‐specific effect, independent of mood or semantic associations. However, it remains unclear whether such suppression extends to tasks involving a higher cognitive load, such as those requiring working memory.

To test whether the effect of limonene could be extended to working memory contexts, we employed a novel 2‐back task using color lightness levels. In the 2‐back task, participants were required to memorize a sequence of stimuli and indicate whether the currently presented stimulus matched that shown in the previous trials. Variants of the N‐back task have been widely used to investigate working memory and related attentional control mechanisms (Owen et al. 2005). The 2‐back task allowed us to examine whether the limonene‐related P300 modulation observed in our previous oddball study would extend to a more demanding memory‐based visual judgment task. Previous studies suggest that parietal P300 amplitude is sensitive not only to selective attention but also to changes in task demand, including working memory load (Guerrero et al. 2022, 2023; Morgan et al. 2008; Ren et al. 2023).

Unlike conventional N‐back tasks that focus on spatial or semantic stimuli, our task manipulates perceptual lightness, which is a rarely used dimension in ERP‐based cognitive studies. This task required participants to memorize and discriminate between different levels of color lightness, thereby engaging higher‐level cognitive functions such as memory storage and change detection. The 2‐back task allowed us to test the reproducibility of the limonene effect and explore how olfactory stimulation might influence task performance under varying cognitive demands. We also tested whether such effects were more pronounced for difficult stimuli (gray) than for easier stimuli (orange).

We hypothesized that limonene would modulate neural processing during the task, particularly under higher cognitive demand, and that such effects might be reflected in P300 responses. By employing a perceptually subtle and cognitively demanding task, we sought to clarify whether the effects of limonene arise from its chemical properties or from broader affective, semantic, or attentional influences.

## 2 Method

### 2.1 Participants

The Ethics Committee of the Fukuoka Institute of Technology approved this experiment in April 2022 (hm01‐22). Participants were prospectively recruited between first May 2023 and November 30, 2023, and between April 10, 2026 and May 28, 2026. Additional participants were recruited during the revision stage to increase statistical reliability and improve gender balance. All participants provided written informed consent in accordance with the tenets of the Declaration of Helsinki.

We recruited 35 university students (14 women, 21 men; mean age ± SD = 21 ± 1.2 years). None of the participants exhibited color vision deficiencies as confirmed via the Ishihara test. Because odor detection and discrimination may vary depending on cultural and linguistic backgrounds, all participants were native Japanese speakers with shared cultural backgrounds. For the ERP analysis, data from one participant were excluded because fewer than 10% of the epochs remained after artifact rejection; thus, data from 34 participants (14 women, 20 men) were analyzed. Because some participants showed extreme hit or false‐alarm proportions in specific conditions, the number of cases included in the SDT‐based analyses varied slightly across measures after applying the standard correction and validity criteria. Although no formal a priori power analysis was conducted, the final sample size was considered sufficient to detect medium to large effects, as supported by the observed Cohen's *f* values and partial η^2^ statistics reported in the Results.

### 2.2 Odor Stimuli

We used (R)‐(+)‐limonene (Fujifilm Wako Pure Chemical Corporation, Osaka, Japan) as the odor stimulus. During the 2‐back task, participants wore a non‐woven mask with a sticker to which the odorous solution (25 µL of limonene) was applied. In the no‐odor condition, the participants wore a mask with a sticker that contained no odorous solution.

### 2.3 2‐Back Color Lightness Task

We implemented a 2‐back color lightness task during odor presentation. The 2‐back task was designed to include two levels of perceptual difficulty by manipulating the lightness of the color stimuli. Color cues were presented randomly, and although the hue values were consistent, the lightness levels varied according to the CIEL*a*b* color space. We used orange and gray to represent low and high cognitive load conditions, respectively. Five lightness levels were prepared for the orange color set (L* = 51.0, 61.0, 71.0, 81.0, and 91.0), all with the same hue (a* = 54.0, b* = 87.4). The gray color set also comprised five lightness levels (L* = 51.0, 61.0, 71.0, 81.0, and 91.0) with a consistent hue (a* = 0.0, b* = 0.0). These colors were selected based on those used in our previous oddball task (Tamura et al. 2025), allowing for consistency across paradigms and facilitating interpretation of ERP effects across different task contexts.

**FIGURE 1:**
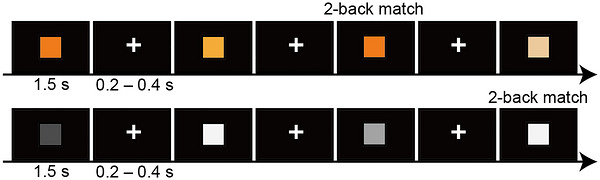
**2‐back color lightness working memory task**. The upper panel indicates the example of a time course using the orange color set, and the lower panel indicates that of the gray color set. Participants were required to memorize the lightness of the colors and respond whether the present lightness level matched that of the two trials prior or not. These sequences were repeated twice for the two odor conditions: no odor and limonene exposure.

Each color cue was presented for 1.5 s, followed by a fixation cross‐presentation for a random duration of 0.2–0.4 s. The participants were allowed to blink their eyes only when a fixation cross was presented. Participants were instructed to memorize the lightness level and respond using a computer mouse: a left‐click if the current lightness level matched that presented in two prior trials and a right‐click if not. Color cues were presented in a randomized order within each color set. Each experimental block consisted of 100 trials (Figure 1).

The participants first completed a 2‐back task block using the orange color set (100 trials), followed by a block using the gray color set. Before and after the 2‐back tests (orange and gray color sets), the participants were asked about subjective odor intensity using a six‐point odor scale ranging from 0 to 5, where 0 = no odor, 1 = barely detectable (detection threshold), 2 = weak but recognizable (recognition threshold), 3 = easily noticeable, 4 = strong, and 5 = extremely strong. These two blocks were repeated twice, corresponding to the odor conditions of no odor and limonene exposure. Between the odor conditions, participants rested for at least 4 min in a ventilated room and were instructed to inhale ambient air by sniffing their own skin to minimize olfactory carryover effects. The order of odor presentation was counterbalanced across participants: they completed the limonene blocks first, followed by the no‐odor blocks, or vice versa.

All visual stimuli were presented on a 24‐inch LCD monitor placed in a darkened room using Psychtoolbox‐3(Brainard 1997; Kleiner et al. 2007) programmed in MATLAB (MathWorks, Inc., Natick, MA, USA). The monitor was calibrated according to standard procedures (Brainard et al. 2002) using a ColorCAL MKII Colorimeter (Cambridge Research Systems Ltd., Rochester, UK).

### 2.4 Electroencephalogram (EEG) Measurement

EEG data were recorded across 19 channels according to the international 10–20 System, using Polymate Pro MP6100 and wet active electrodes (Miyuki Giken Co., Ltd., Tokyo, Japan). The ground electrode was placed at Fpz, and the reference electrode was A1. The data were amplified and digitized at a sampling rate of 500 Hz, with impedance kept below 10 kΩ.

In the offline analysis, the data were referenced to the average of electrodes A1 and A2. Signals were bandpass‐filtered between 0.1 and 50 Hz using an FIR filter. Epochs were rejected if the amplitude exceeded ±80 µV within the −200 to 800 ms window. Epochs were segmented relative to visual stimulus onset (time 0 ms) and averaged for each participant. Baseline correction was applied using a −200 to 0 ms interval. Preprocessing and averaging were performed using EEGLAB (Delorme and Makeig 2004) and ERPLAB (Lopez‐Calderon and Luck 2014).

### 2.5 Memory Performance

Memory performance in the 2‐back task was analyzed using signal detection theory (SDT). We calculated the correct response rate, the sensitivity index (*d*’), and the response bias (*C*).

In this study, a “Hit” was defined as correctly identifying a lightness level that matched the one presented two trials earlier. A “false alarm” was defined as incorrectly responding “same” when the lightness level did not match the one presented two trials earlier.

The sensitivity index *d*’ was computed using the standard SDT formula:

$$\;d^{\prime }=Z\left(p_{H}\right)-Z\left(p_{FA}\right),$$

where Z(p) indicates the inverse of the cumulative normal distribution function, $p_{H}$ and $p_{FA}$ represent the ratio of hit rate and false‐alarm rate, respectively.

Hit rate was calculated as:

$$\;p_{H}=\frac{N_{Hits}}{TN_{target}},$$

where $N_{Hits}$ indicates the number of hits, and *TN_target_* indicates the total number of target‐presented trials. The false‐alarm rate was calculated as follows:

$$\;p_{FA}=\frac{N_{FA}}{TN_{absent}}.$$

where $N_{FA}$ indicates the number of false alarms, and *TN_absent_* indicates the total number of target‐absent trials.

Response bias *C* indicates the tendency of the participants to respond “same” or “different” regardless of the actual lightness. *C* = 0 indicates no bias; *C* > 0 suggests a bias toward responding “different,” and *C* < 0 suggests a bias toward responding “same.” *C* was calculated as:

$$C=-\;\frac{Z\left(p_{H}\right)+Z\left(p_{FA}\right)}{2}.$$

### 2.6 Statistics

The correct response rate, sensitivity index (*d*’), and response bias (*C*) of the SDT were assessed using two‐way analysis of variance (ANOVA) for within‐subject analysis (odor condition × color set). The peak amplitude of P300 within the 250–420 ms was also assessed using a two‐way ANOVA for within‐subject analysis (odor condition × color set) at the Cz and Pz electrodes. For post hoc testing, we performed simple main effects tests to compare peak amplitudes between the two color sets under each odor condition. The tests were conducted using JMP Pro 18(SAS Institute Inc., Cary, NC, USA). Effect sizes were calculated using Cohen's *d* or *f* according to Cohen's criteria (Cohen 1988). Post hoc power analysis for the primary ERP interaction effect was estimated from the observed effect sizes (Cohen's *f*) at α = 0.05.

## 3 Results

### 3.1 Memory Performance

**FIGURE 2:**
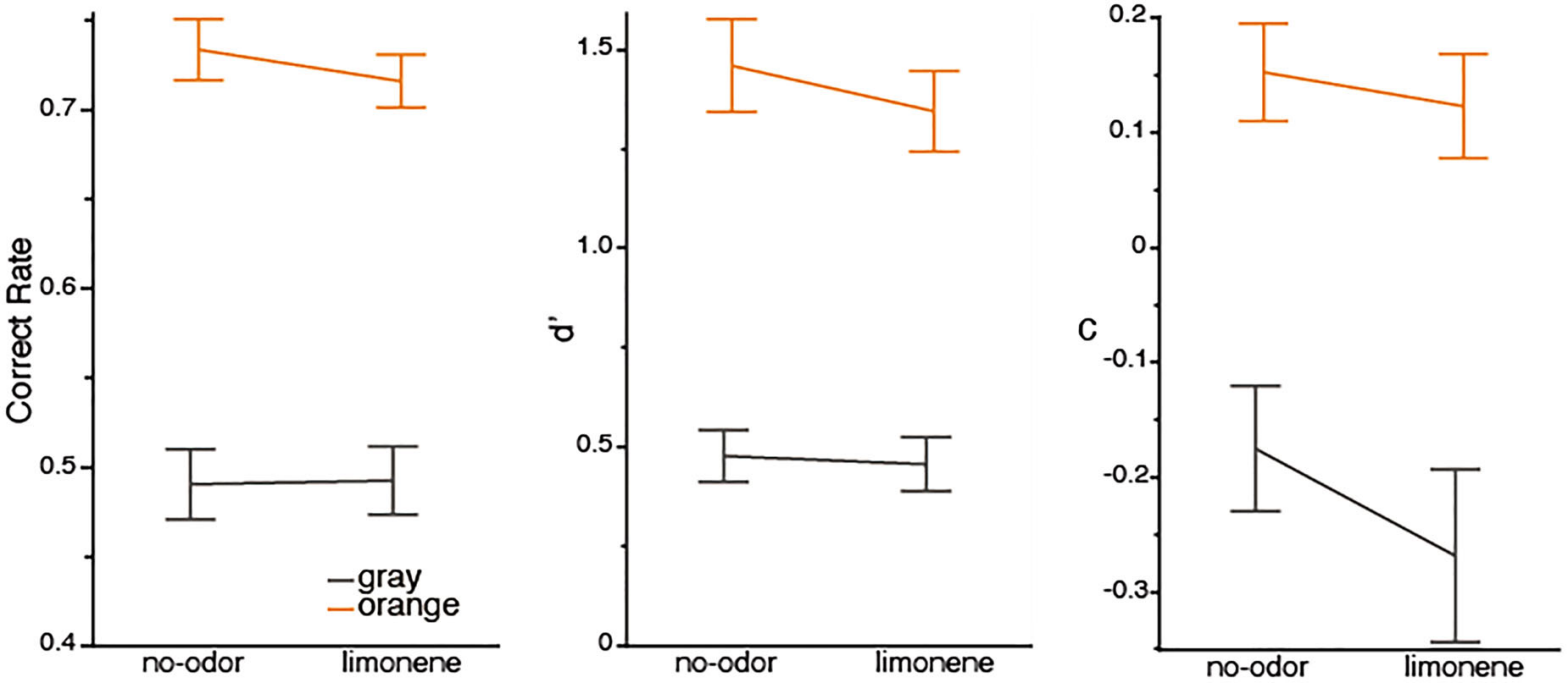
**Memory performance in the 2‐back lightness working memory task**. The line plots show the correct response rate, sensitivity index (*d*’), and response bias (*C*) under the limonene exposure and no‐odor conditions, separated by color set (orange and gray). Error bars represent standard errors. Performance differed between color sets, with higher accuracy, sensitivity, and response bias for orange than gray stimuli.

The two‐way ANOVA revealed a significant main effect of color set: working memory performance was better for orange lightness stimuli than for gray stimuli (correct rate: *F*(1,33) = 167, *p* < 0.0001, partial η^2^ = 0.84, Cohen's *f* = 2.3; *d*’: *F* (1,32) = 136, *p* < 0.0001, partial η^2^ = 0.81, Cohen's *f* = 2.1; *C*: *F*(1,30) = 25.9, *p* < 0.0001, partial η^2^ = 0.46, Cohen's *f* = 0.93). These results indicate that the participants performed better with orange than gray stimuli in the lightness working memory task (Figure 2). Descriptive statistics for all behavioral measures are presented in Table 1.

**TABLE 1:** Mean and standard deviation of working memory performance using SDT.

		Correct rate	Hit rate	False‐alarm rate	*d*’	*C* (response bias)
Orange	No‐odor	0.73 ± 0.10	0.69 ± 0.16	0.20 ± 0.086	1.5 ± 0.69	0.15 ± 0.25
	Limonene	0.72 ± 0.088	0.70 ± 0.16	0.22 ± 0.081	1.3 ± 0.59	0.13 ± 0.26
Gray	No‐odor	0.49 ± 0.12	0.67 ± 0.16	0.50 ± 0.18	0.48 ± 0.38	0.48 ± 0.31
	Limonene	0.49 ± 0.11	0.68 ± 0.16	0.52 ± 0.18	0.46 ± 0.40	0.46 ± 0.44

For the main effect of odor, there was no significant effect on correct rate, sensitivity index (*d*′), or response bias (*C*) (correct rate: *F*(1,33) = 0.74, *p* = 0.40, partial η^2^ = 0.022, Cohen's *f* = 0.15; *d*’: *F*(1,32) = 0.80, *p* = 0.38, partial η^2^ = 0.024, Cohen's *f* = 0.15; *C*: *F*(1,34) = 3.0, *p* = 0.091, partial η^2^ = 0.081, Cohen's *f* = 0.30). There was no significant interaction effect between odor and color set (correct rate: *F*(1,33) = 0.63, *p* = 0.43, partial η^2^ = 0.019, Cohen's *f* = 0.14; *d*’: *F*(1,32) = 0.20, *p* = 0.65, partial η^2^ = 0.006, Cohen's *f* = 0.079; *C*: *F* (1,26) = 0.13, *p* = 0.71, partial η^2^ = 0.005, Cohen's *f* = 0.071).

### 3.2 ERP

**FIGURE 3:**
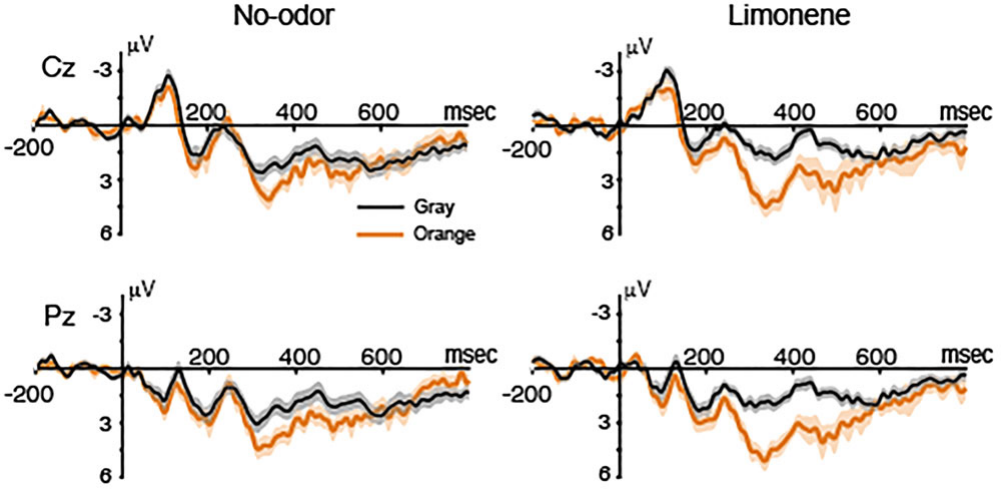
**ERP waveforms at the Cz and Pz electrodes during the 2‐back task**. Grand average ERP waveforms at Cz (upper) and Pz (bottom) under limonene and no‐odor conditions, separated by color set (orange and gray). Shaded areas represent standard errors. The P300 peak was determined within the 250–420 ms window.

ERP analyses were performed using epochs time‐locked to memory cue onset. The P300 peaks evoked by the 2‐back lightness task were generally larger for orange stimuli than for gray stimuli, with a stronger differentiation under the limonene exposure condition, within 250–420 ms (Figure 3). Cz and Pz were selected as the main electrodes of interest.

At the Cz electrode, there was a significant main effect of color sets (*F*(1,33) = 20, *p* < 0.0001, partial η^2^ = 0.38, Cohen's *f* = 0.77, post hoc power > 0.99), but no significant main effect of the odor condition (*F*(1,33) = 0.19, *p* = 0.66, partial η^2^< 0.01, Cohen's *f* = 0.077, post hoc power = 0.072). There was a significant interaction effect between odor conditions and color sets (*F*(1,33) = 10, *p* = 0.0028, partial η^2^ = 0.24, Cohen's *f* = 0.56). Simple main effects tests showed significantly lower P300 amplitude in the limonene condition for the gray color set (*F*(1,61) = 5.2, *p* = 0.026, partial η^2^ = 0.079, Cohen's *f* = 0.29). There was no significant difference between the odor conditions for the orange color set (*F*(1,61) = 2.5, *p* = 0.12, partial η^2^ = 0.039, Cohen's *f* = 0.20).

**FIGURE 4:**
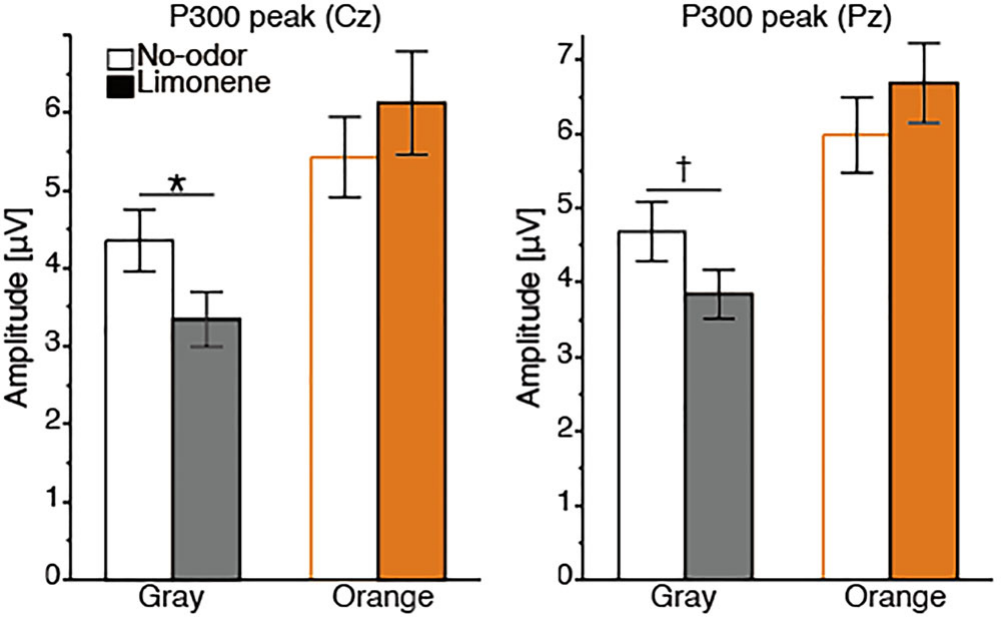
**Mean P300 peak amplitudes at the Cz and Pz electrodes in the 2‐back task**. Mean P300 amplitudes (250–420 ms) at the Cz (left) and Pz (right) electrodes under the limonene exposure and no‐odor conditions, separated by color set. Error bars indicate standard errors. A significant interaction was found between odor condition and color set, with post hoc tests revealing significantly lower P300 amplitudes in the limonene condition than in the no‐odor condition for gray stimuli at Cz, whereas a marginal difference was observed at Pz. *p < 0.05, †*p* < 0.10.

At the Pz electrode, there was a significant main effect of color sets (*F*(1,33) = 29, *p* < 0.0001, partial η^2^ = 0.47, Cohen's *f* = 0.94, post hoc power > 0.99), but no significant main effect of the odor condition (*F*(1,33) = 0.037, *p* = 0.85, partial η^2^ = 0.0011, Cohen's *f* = 0.034, post hoc power = 0.054). There was a significant interaction effect between odor conditions and color sets (*F*(1, 33) = 12, *p* = 0.0014, partial η^2^ = 0.27, Cohen's *f* = 0.61). Simple main effects test showed lower P300 in the limonene condition for the gray color set, but did not reach statistical significance (*F*(1,54) = 3.9, *p* = 0.055, partial η^2^ = 0.066, Cohen's *f* = 0.27). There was no significant difference between the odor conditions for the orange color set (*F*(1,54) = 2.65, *p* = 0.11, partial η^2^ = 0.047, Cohen's *f* = 0.22) (Figure 4). A post hoc power analysis indicated that the odor × color‐set interaction in the ERP analysis was adequately powered (achieved power: Cz = 0.89, Pz = 0.93).

### 3.3 Subjective Odor Intensity

Subjective odor intensity ratings, assessed before and after each task block using the six‐point scale described in the Methods, are presented in Table 2.

**TABLE 2:** Subjective odor intensity of odor conditions.

Odor	Before (*M* ± SD)	After (*M* ± SD)	Mean Diff	*t*	*p*	Cohen's *d*
Limonene	3.03 ± 0.68	2.38 ± 1.00	−0.64	−4.27	0.0002	0.73
No‐odor	0.79 ± 0.98	0.71 ± 0.97	−0.09	−1.00	0.32	0.17

*Note*: Mean subjective odor intensity ratings before and after each experimental block under different odor conditions. Ratings were assessed on a six‐point scale from 0 (no odor) to 5 (extremely strong odor). Ratings before and after each experimental block were compared using paired *t*‐tests.

## 4 Discussion

This study investigated whether limonene, a principal volatile compound in citrus peel, modulates cognitive processing during a visual 2‐back working memory task under different levels of perceptual load. Building on our previous finding that limonene suppresses P300 amplitude during a low‐demand visual oddball task (Tamura et al. 2025), we developed a 2‐back task using color lightness discrimination to examine whether this modulatory effect would extend to a task imposing higher cognitive demand. The main findings were that (1) P300 amplitudes showed a load‐dependent modulation with a significant interaction between odor condition and stimulus type, and (2) behavioral performance differed depending on stimulus difficulty but was not significantly affected by limonene.

The behavioral analysis did not reveal significant effects of limonene on correct response rate, sensitivity (*d*′), or response bias (*C*), indicating that limonene did not measurably alter overt task performance under the present experimental conditions. Instead, the behavioral results were primarily determined by stimulus difficulty: performance was better for orange than for gray stimuli, suggesting that the gray condition imposed greater task demands. These findings, therefore, indicate that any effect of limonene was subtle and was not reflected in the behavioral indices used in the present study.

In contrast to the behavioral results, the ERP analyses revealed that P300 amplitudes were more strongly reduced for gray than for orange stimuli under limonene exposure, resulting in a significant odor × color‐set interaction. This P300 decrease may therefore be interpreted as a consequence of the differential cognitive demands imposed by the two color conditions. This dissociation between neural and behavioral measures suggests that limonene may have influenced underlying cognitive processing even when overt task performance remained unchanged. Similar dissociations have been reported in studies showing that electrophysiological indices can be sensitive to task demand or resource allocation even when behavioral measures show weaker or no corresponding effects. For example, P300 reduction has been observed under higher workload (Käthner et al. 2014) and under a demanding dual‐task manipulation without corresponding differences in some behavioral indices (Kida et al. 2004).

Importantly, the P300 component should not be interpreted as a marker of working memory processes alone. Polich (2007) characterized P3a and P3b as components linked to attention, stimulus evaluation, and memory‐related operations, rather than to a single cognitive function in isolation. In addition, it has been proposed that the classic P300 can reflect a build‐to‐threshold decision variable, suggesting that P300 may index decision formation as well as attentional processing (Twomey et al. 2015). This broader interpretation is also consistent with recent work using the n‐back paradigm, which emphasizes that the task progressively taxes the attentional system in addition to its working memory demands (Pereira Soares et al. 2024). Moreover, Li et al. (2024) suggested that, within the n‐back task, reduced P300 amplitude may be particularly related to updating‐related processing under higher memory load, whereas slow‐wave activity may be more closely associated with maintenance. Scharinger et al. (2017) further showed that P300 amplitude differed across working memory paradigms, with particularly pronounced effects in the n‐back task, which they interpreted as reflecting specific cognitive control demands. The present findings should therefore be interpreted within this broader framework, rather than as evidence for a selective modulation of working memory itself.

One possible interpretation is that the reduced P300 amplitude reflects decreased attentional resource allocation under higher task demand. In our previous study, we proposed that the P300 reduction under limonene exposure was related to inhibition of selective attention processes in a classic oddball task (Tamura et al. 2025). This interpretation was consistent with the report that P300 amplitude decreased with increasing memory load in an n‐back task (Watter et al. 2001). Likewise, P300 reduction has been reported with increasing working memory load (Morgan et al. 2008; Ren et al. 2023). Taken together, these studies support the interpretation that lower P300 amplitude in the present gray condition may reflect reduced availability of attentional resources under more demanding task conditions.

At the same time, not all studies support a simple monotonic decrease in P300 across all contexts, and some findings point instead to a redistribution of frontal and parietal activity under increasing task demand. It has been reported that higher task difficulty was associated with reduced parietal P300 amplitude, whereas frontal P300 changed differently across age groups (Guerrero et al. 2022), suggesting that neural responses to load may reflect changes in neural capacity and compensatory recruitment rather than uniform amplitude reduction. Similarly, another P300 study of working memory reported that increasing task load reduced parietal‐over‐frontal predominance mainly because of greater frontal recruitment, linking P300 topography to working memory capacity (Guerrero et al. 2023). These findings suggest that the present central‐parietal P300 effect is compatible with load‐related modulation but should not be interpreted too narrowly as a purely working‐memory‐specific signal.

A further possibility is that the present P300 modulation reflected changes in decision‐related processing. Twomey et al. (2015) proposed that the classic P300 reflects the accumulation of evidence toward a decision bound, reaching a stereotyped amplitude before response execution and varying with task difficulty and reaction time. From this perspective, the reduced P300 amplitude observed in the gray condition may reflect less efficient or less stable evidence accumulation during more difficult discriminations. Under this account, limonene may have modulated the efficiency of decision‐related processing under higher task demand, rather than memory storage itself. This framework may be especially relevant in the present task, in which participants were required not only to maintain color lightness information but also to judge whether the current stimulus matched the stimulus presented two trials earlier.

P300 amplitudes were examined at central (Cz) and parietal (Pz) sites to facilitate comparison with our previous study (Tamura et al. 2025), which reported P300 suppression by limonene during a visual oddball task. Extending this work to a more demanding memory‐based task, we observed a load‐dependent modulation of P300, with reduced amplitudes under limonene exposure specifically in the gray (high‐load) condition. In contrast, no such modulation was observed for the orange (low‐load) condition. The present findings indicate that this modulation should not be interpreted as reflecting a selective effect on working memory itself, but rather within a broader framework in which P300 reflects multiple cognitive processes, including attentional allocation, task demand, and decision‐related processing.

The present findings can also be situated within a broader literature showing that olfactory input can influence visual cognition at multiple processing stages. At an early perceptual stage, Tsushima et al. (2021) demonstrated that odor can modulate visual motion perception and alter activity in visual cortical areas even without prior training. At a higher level, Rekow et al. (2022) showed that odor selectively enhanced the neural categorization of ambiguous face‐like visual stimuli, suggesting that olfactory effects may become especially apparent when visual input is uncertain or difficult to interpret. Similarly, Hörberg et al. (2020) reported that incongruent odors interfered with visual decisions and were associated with a late slow‐wave ERP effect, indicating that odor can also influence later categorization or decision‐related stages of processing. Extending this work to demanding goal‐directed vision, it has been shown that congruent odors improved performance and speeded responses in a challenging visual search task (Castellotti et al. 2025). In this broader context, the classic study by Gottfried and Dolan (2003) provides an important framework for understanding such findings by demonstrating cross‐modal integration between olfactory and visual information. Together, these studies suggest that odor‐related modulation of visual cognition is not limited to a single stage of processing, but may vary depending on task demands, stimulus ambiguity, and cross‐modal congruency.

Previous studies suggest that odor‐related effects on cognition can vary depending on odor composition, task structure, and cognitive load. A related study (Ueda et al. 2023) reported that lemon essential oil enhanced performance in a 2‐back letter working memory task, but its experimental design differed substantially from the present one in at least two important respects. That study examined lemon essential oil, which contains multiple compounds in addition to limonene, rather than pure limonene. They also assessed performance before and after inhalation rather than during continuous exposure in the task itself. Thus, the discrepancy between their findings and the present results may reflect differences in odor composition, task structure, and timing of exposure rather than a direct contradiction. In another relevant study, it has been shown that high working memory demand can reduce the behavioral impact of aversive olfactory distraction, with prefrontal mechanisms identified as underlying this buffering effect (Weigard et al. 2021). Tarrida et al. (2025) showed that even brief odor cues can induce sustained neural activity beyond odor offset and influence later memory‐related processing, highlighting the possibility that olfactory effects can persist beyond odor offset and shape subsequent cognitive processing.

One limitation of the present study is that only a single odorant was compared with a no‐odor condition. Therefore, it was not possible to fully distinguish limonene‐specific effects from the general presence of odor. To clarify odor‐specific influences on cognitive processes, future studies should include additional odorants.

Another important consideration concerns the potential effects of task order. In the present task sequence, odor condition order was counterbalanced, but the color condition was fixed. We observed a significant decline in P300 amplitude for the gray color set, which was completed after the orange set. The fixed design could not reject the possibility that the apparent increase in demand for the gray color set may have been partly influenced by fatigue. We tried to maintain a certain odor intensity; however, the subjective ratings of perceived odor intensity were decreased after the experiment, maybe due to olfactory habituation. The P300 at gray‐color sets under the limonene presentation may have been reduced by the confounding of the limonene effect, fatigue, and habituation. Better habituation control and a modified task design would help clarify the limonene‐specific effect in future studies. Furthermore, the inclusion of reaction time measures would help disentangle the relative contributions of working memory demands and olfactory input.

## 5 Conclusion

This study suggested that limonene modulates neural processing during a demanding visual 2‐back task, with its effects being most evident in the P300 response under higher task demand. Building on our previous finding that limonene reduced P300 amplitude in a visual oddball task (Tamura et al. 2025), the present results extend this observation to a task requiring sustained memory‐based visual judgments. Importantly, limonene did not significantly affect behavioral measures of task performance, indicating that its influence was more clearly reflected in neural activity than in overt responses. Taken together, these findings support the view that limonene may influence neural processes associated with attentional allocation and/or decision‐related processing under load, rather than selectively altering working memory itself. Future studies should employ fully counterbalanced designs, include additional odor conditions, and incorporate reaction time measures to clarify the mechanisms underlying odor‐related modulation of cognition.

## Author Contributions

**Toko Tatsumoto**: investigation. Yuko Ohno: conceptualization, supervision, Writing – review and editing. **Taiki Nishimura**: data curation, formal analysis, investigation. **Takumi Yano**: investigation. **Mitsuo Tonoike**: conceptualization, supervision, writing – review and editing. **Kaori Tamura**: conceptualization, methodology, formal analysis, funding acquisition, writing – original draft, writing – review and editing, supervision. **Keitaro Yamashita**: investigation, data curation, formal analysis. **Shin'ichi Yoshimura**: conceptualization, supervision, writing – review and editing.

## Funding

This study was supported by a Grant‐in‐Aid for Scientific Research (KAKENHI) from the Japan Society for the Promotion of Science (Grant Number 23K11297).

## Conflicts of Interest

The authors declare no conflict of interest

## Declaration of Generative AI and AI‐Assisted Technologies in the Manuscript Preparation Process

During the preparation of this work, the author used ChatGPT (GPT‐4o, OpenAI) to improve English language clarity during manuscript preparation. AI was not used to generate the original content, data analysis, or programming. All scientific reasoning, analyses, and interpretations were conducted solely by the authors. After using this tool/service, the authors reviewed and edited the content as needed and take full responsibility for the content of the published article.

## Data Availability

The datasets collected and analyzed in the current study are not publicly available because of ethical restrictions and participant confidentiality, as approved by the institutional review board. The analytical code is available from the corresponding author upon reasonable request.
